# Combining Dynamic Stretch and Tunable Stiffness to Probe Cell Mechanobiology *In Vitro*

**DOI:** 10.1371/journal.pone.0023272

**Published:** 2011-08-15

**Authors:** Angela M. Throm Quinlan, Leslie N. Sierad, Andrew K. Capulli, Laura E. Firstenberg, Kristen L. Billiar

**Affiliations:** 1 Department of Biomedical Engineering, Worcester Polytechnic Institute, Worcester, Massachusetts, United States of America; 2 Graduate School of Biomedical Sciences, UMass Medical School, Worcester, Massachusetts, United States of America; 3 Franklin W. Olin College of Engineering, Needham, Massachusetts, United States of America; 4 Department of Surgery, University of Massachusetts Medical School, Worcester, Massachusetts, United States of America; RMIT University, Australia

## Abstract

Cells have the ability to actively sense their mechanical environment and respond to both substrate stiffness and stretch by altering their adhesion, proliferation, locomotion, morphology, and synthetic profile. In order to elucidate the interrelated effects of different mechanical stimuli on cell phenotype *in vitro*, we have developed a method for culturing mammalian cells in a two-dimensional environment at a wide range of combined levels of substrate stiffness and dynamic stretch. Polyacrylamide gels were covalently bonded to flexible silicone culture plates and coated with monomeric collagen for cell adhesion. Substrate stiffness was adjusted from relatively soft (G′ = 0.3 kPa) to stiff (G′ = 50 kPa) by altering the ratio of acrylamide to bis-acrylamide, and the silicone membranes were stretched over circular loading posts by applying vacuum pressure to impart near-uniform stretch, as confirmed by strain field analysis. As a demonstration of the system, porcine aortic valve interstitial cells (VIC) and human mesenchymal stem cells (hMSC) were plated on soft and stiff substrates either statically cultured or exposed to 10% equibiaxial or pure uniaxial stretch at 1Hz for 6 hours. In all cases, cell attachment and cell viability were high. On soft substrates, VICs cultured statically exhibit a small rounded morphology, significantly smaller than on stiff substrates (p<0.05). Following equibiaxial cyclic stretch, VICs spread to the extent of cells cultured on stiff substrates, but did not reorient in response to uniaxial stretch to the extent of cells stretched on stiff substrates. hMSCs exhibited a less pronounced response than VICs, likely due to a lower stiffness threshold for spreading on static gels. These preliminary data demonstrate that inhibition of spreading due to a lack of matrix stiffness surrounding a cell may be overcome by externally applied stretch suggesting similar mechanotransduction mechanisms for sensing stiffness and stretch.

## Introduction

Proper spatiotemporal distributions of dynamic physical cues are necessary to guide the development, maintenance, and healing of tissues. Cells such as fibroblasts, endothelial cells, and muscle cells actively sense both the external loading applied to them (outside-in signaling) and the stiffness of their surroundings (inside-out signaling). They respond to these stimuli with changes in adhesion, proliferation, locomotion, morphology, and synthetic profile (reviewed in [1], [2]). Although some likely candidates for sensing stiffness and stretch exist, it remains unclear if the same mechanotransduction pathways are responsible for inside-out and outside-in signaling, or if there are mechanosensing and mechanoregulation machinery specific to each stimulus. A better understanding of how complex combinations of mechanical stimuli regulate cell behavior is critical for the rational engineering of tissues *in vitro* and for guiding proper regeneration *in vivo*.

Leung et al. [3] first described the sensitivity of cells to dynamic stretch *in vitro* by demonstrating a change in protein production in equibiaxially cycled smooth muscle cells, and subsequent studies have demonstrated that mechanical stretching induces a wide range of cellular responses including cytoskeletal remodeling, synthesis of numerous extracellular matrix proteins, and altered expression of a multitude of genes [4], [5]. Cell reorientation “away” from the direction of maximal cyclic stretch is the most visible effect of stretch and is accompanied by pronounced remodeling of the actin cytoskeleton [6], [7]. *In vitro* investigations into the role of stretch on cell behavior are most commonly carried out on protein-coated silicone substrates. Countless custom loading devices have been developed for both uniaxial [8] and biaxial [9] stretch patterns. Commercial devices are also available such as Flexcell®, which uses vacuum pressure to stretch a circular silicone membrane over a fixed loading post, and STREX which utilizes dual motors to stretch square or rectangular wells biaxially. As cells are not able to appreciably deform the relatively stiff silicone substrates used in standard cell-stretch systems (Young's modulus≈150 kPa), it is not possible to quantitatively investigate the effects of stretch on the traction forces the cells exert on the substrate or to determine the effect of substrate stiffness (and resulting prestress) on the cellular response to stretch.

Cells are influenced by the stiffness of their surroundings and exert tension on their environment, a phenomena first described by Harris [10] with cells wrinkling the membrane on which they were cultured. Since that time, it has been clearly shown that the stiffness of the culture environment is a potent stimulus for a variety of cell functions. Stiffness induces wide-ranging effects on cell behavior, the most obvious being spread area and level of prestress. For example, fibroblasts cultured on soft substrates (E≈1 kPa) have significantly smaller spread area and shape factor than those cultured on stiff substrates (e.g., glass, E≈1 GPa) [11]. Changes in cytoskeletal organization [12], matrix adhesions [11], migration, growth [13], maturation [14], contractile force generation [15], and myofibroblast differentiation [16] have also been reported. Recent studies indicate that stem cell differentiation can be guided by stiffness [17], [18]. *In vitro* investigations into the role of stiffness on cell behavior are most commonly carried out on two-dimensional (2D) polyacrylamide (PA) substrates by changing the polymer chemistry to alter the substrate stiffness as described in the work of Y-L Wang and colleagues [19], although other polymer systems have also been utilized both in 2D and 3D configurations, e.g., polyethylene glycol (PEG) [20] and polydimethyl siloxane (PDMS) [10]. Cellular deformation of these compliant substrates has also been exploited to quantify the forces that the cell exerts on the substrate utilizing powerful traction force microscopy techniques [21].

Recently, Fredberg and colleagues [22] developed an indenter-based method (termed “Cell Mapping Rheometry, CMR”) to locally deform single cells cultured on soft PA substrates. The authors probed the time-course of changes in cell traction forces following single and multiple cycles of biaxial and uniaxial stretch and demonstrated cytoskeletal fluidization or reinforcement in response to uniform and non-homogeneous strain fields, respectively. In its current configuration, CMR is ideal for the study of single cells in short duration studies of the dynamics of traction forces and cytoskeletal stiffness. However, a larger format system for combining levels of stretch and stiffness would be of benefit for elucidating mechanotransduction pathways requiring large numbers of cells for gene and protein regulation analyses, and for cell differentiation studies requiring long culture duration.

The goal of this work is to develop an *in vitro* method to investigate the combined role of substrate stiffness and dynamic stretch on cell behavior. Due to common pathways reported for outside-in (stretch-induced) and inside-out (stiffness-induced) cell signaling, we hypothesize that the application of cyclic stretch to cells cultured on soft hydrogels will induce responses commonly observed in cells cultured on stiff substrates. From the many possible means of controlling substrate stiffness and membrane stretch, we chose to covalently bind PA, the most common “tunable” stiffness substrate, to a widely used dynamic cell culture substrate available commercially (Bioflex Culture Plates, Flexcell International) to ensure that the method could be implemented widely. Although seemingly a straightforward approach, the tight control of the process variables necessary for robust linkage of the PA to the silicone membrane required for large amplitude dynamic deformation has been a common stumbling block. To verify that the strain field presented to the cells by the silicone membrane is not altered by the thin PA gel, we utilize High Density Mapping (HDM) analysis. As a demonstration of the utility of this method we examine the spreading behavior of adherent valvular and stem cells using these mechanical stimuli in concert; most notably we investigate initially rounded cells on very soft substrates subjected to equibiaxial stretch and report a novel outcome. Implications of our preliminary results are discussed along with potential future investigations made possible with the method described herein.

## Methods

### Culture Plate Preparation

PA gels of defined stiffness levels were chemically attached onto standard 6-well flexible silicone membranes. To facilitate attachment, untreated Bioflex Culture Plates (Flexcell International) were functionalized using a protocol modified from that of Silver et al. [23]. The plates were oxygen plasma treated for 2 minutes (Plasma Prep II, SPI) and then immediately treated with 4.7 mM 3-(Trichlorosilyl)propyl methacrylate (Sigma) in a 4∶1 solution of heptanes and carbon tetrachloride for five minutes, after which the solution was removed and the silicone was rinsed with hexane. The plates were transferred to a vacuum chamber and negative pressure was applied for five minutes to remove volatile solvents from the silicone. The vacuum was released from the vacuum chamber and the chamber was flushed with nitrogen gas. STREX chambers (10 cm^2^, B-Bridge International, Inc.) with flexible silicone culture surfaces were also treated with the above protocol for comparison.

**Figure 1:**
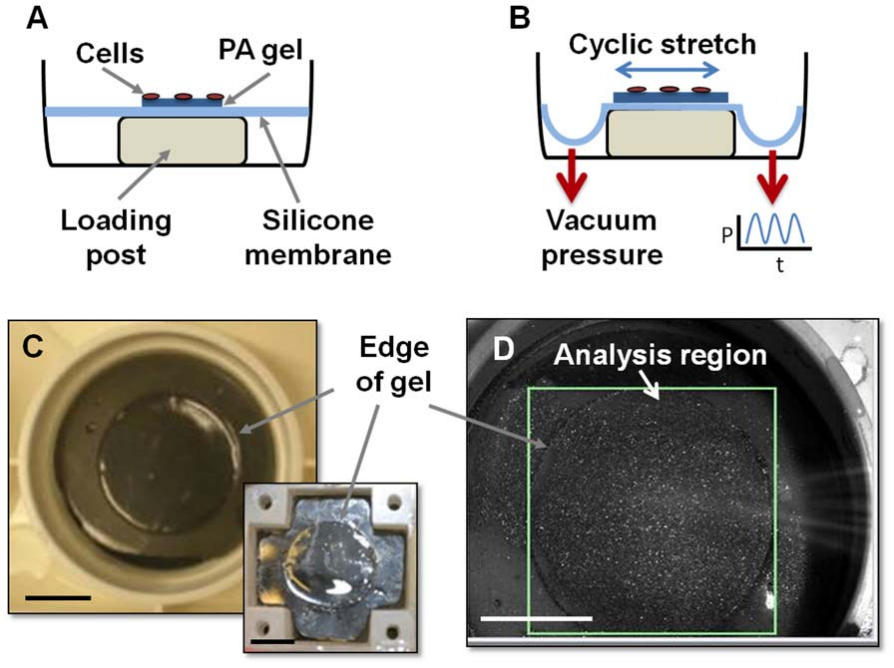
Schematic of polyacrylamide gel on flexible silicone membrane under static (A) and stretched (B) conditions. Top view of a 22 mm diameter collagen-coated gel (∼70 µm thickness) is cast into a 35 mm diameter flexible-bottomed Flexcell™ well (C) and STREX well (C, insert). Image of Flexcell™ well (D) stretched above an Arctangle™ loading post and labeled with retroreflective beads for strain field analysis. Rectangle shows region analyzed in HDM software, arrows point to edge of gel. Scale bars = 10 mm in all panels.

Collagen-coated polyacrylamide substrates were prepared based on standard protocols using a hetero-bifunctional UV activated crosslinker [19] adapted to the silicone-bottomed flexible well format (Figure 1). Briefly, 50 µL of a polyacrylamide gel solution consisting of 0.15% tetramethylethylenediamine, 0.075% ammonium persulfate, and acrylamide∶bisacrylamide (all from Biorad) of varied ratio (Table 1) to control stiffness was applied to the center of each well. Coverslips (22 mm diameter) were made hydrophobic to prevent adhesion to the gels by treating with undiluted Surfacil (Pierce) for one minute and then rinsing with methanol. The treated coverslips were placed on top of the unpolymerized gel solution and left undisturbed until gel polymerization (under nitrogen flow) after which they were removed. The photo-activatable, heterobifunctional cross-linker, sulfosuccinimidyl 6 (4-azido-2-nitrophenyl-amino)hexanoate) (Sulfo-SANPAH, Thermo Scientific) was applied to the surface of each gel and activated with UV light as previously described [24] and 100 µg/mL type I collagen (Purecol, Advanced Biomatrix) was applied to the surface of each gel and incubated for four hours at room temperature. Gels were rinsed with PBS and UV sterilized prior to cell seeding.

**Table 1:** Average strain (± SD) within central region used for analysis of cell morphology for equibiaxial stretch (round loading post) and uniaxial stretch (Arctangle™ loading post).

Stretch	Gel Stiffness	Average Strain
Equibiaxial	0.3 kPa	9.3±0.4%
	50 kPa	7.9±0.6%
	No gel	11.1±0.6%
Uniaxial	0.3 kPa	10.9±0.6%
	50 kPa	7.83±0.3%
	No gel	9.1±0.9%

### Polyacrylamide gel stiffness

The bulk stiffness of the gels was measured by oscillatory shear rheometry using an AR-G2 rheometer (TA Instruments). A volume of 155 µl of polyacrylamide solution was placed on the Peltier plate of the rheometer and a 40 mm diameter parallel plate geometry was lowered to a gap of 100 µm. After polymerizing for 10 minutes, 1× PBS was added around the circumference of the testing geometry to minimize drying, and the temperature was brought to 37°C. Following a 1 Hz 0.1% strain-controlled time sweep to monitor PA polymerization, a 1 Hz stress sweep between 10 and 1000 Pa was performed with the normal force held at 1 N, and the storage modulus (G′) and loss modulus (G″) were measured. Three measurements were made on each gel, gels were measured in duplicate, and values were averaged. As G″ was over an order of magnitude lower than G′, the gels were considered elastic. A wide range of acrylamide∶bisacrylamide combinations were tested and two formulations were utilized for subsequent cell culture experiments: one low stiffness (3% acrylamide, 0.058% bisacrylamide, G′ = 0.3 kPa) and one high stiffness (7.5% acrylamide, 0.117% bisacrylamide, G′ = 50 kPa).

### Polyacrylamide gel stretch validation

Samples were marked with silicon carbide particles (40 µm diameter) and retro-reflective beads (60 µm diameter) to create a random light intensity distribution in the region of interest (ROI) and stretched to 10% using the Flexcell FX-4000T system (Flexcell International, Figure 1). Digital images were acquired at a rate of 50 frames per second using a 1280×1024 pixel resolution CMOS camera (Photron Model # Fastcam-X 1280 PCI) with an 8 bit pixel depth while the Bioflex plates were cycled at 1 Hz from 0 to 10% strain. The strain distributions across the stretched samples were evaluated using digital image analysis. Specifically, the components of the two-dimensional deformation field (*u*1 and *u*2 along the *X*1 and *X*2 camera axes, respectively) were determined from the images by measuring light distribution patterns using High-Density Mapper (HDM) software [25]. In brief, HDM converts the light distribution to the spectral domain using a fast Fourier transform (FFT) and through the use of an interference function, the displacement and rotation are found. The displacements are then converted back from the spectral domain to Cartesian coordinates using an inverse FFT. The chosen field of view (FOV) resulted in a camera resolution of 0.02 mm/pixel. Displacements were measured using a 1.28 mm (64 pixel) subimage size with a corresponding step size of 0.64 mm (32 pixel shift) yielding a 25×20 matrix of *u*1 and *u*2 values for a ∼16×∼13 mm ROI.

### Cell Culture

Valvular interstitial cells (VICs) were isolated [26] from porcine tissue samples obtained from a local abattoir (Blood Farm, Groton, MA) or from the University of Massachusetts Medical School Department of Animal Medicine, from the carcasses of recently euthanized animals that had been used in other, non-related, animal studies, ***which had appropriate IACUC approval***. Once the animals are euthanized, use of the carcasses and tissues are no longer covered by the IACUC and, thus, the tissue harvest process has no protocol number associated with it. The aortic valve was excised and rinsed in 1× phosphate buffered saline. The valve leaflets were incubated in a 600 U/mL solution of Type II collagenase (Worthington Biochemical) in Dulbecco's Modified Eagle's Medium (DMEM, Mediatech) with 100 U/mL penicillin G sodium (Sigma), 100 µg/mL streptomycin sulfate, and 250 ng/mL amphotericin B (Invitrogen) for 20 min on a rocking platform in a 37°C incubator. After incubation, the surface of the valves were scraped with a cell scraper to remove endothelial cells, rinsed in sterile 1× PBS (Mediatech), and cut into 1 mm pieces with a scalpel. The valve pieces were incubated in a fresh 600 U/mL collagenase solution as described above for 2 hr on a rocking platform in a 37°C incubator. The resulting cell/tissue solution was filtered through a nylon mesh, pelleted, and resuspended in standard medium (DMEM, 100 U/mL penicillin G sodium, 100 µg/mL streptomycin sulfate, and 250 ng/mL amphotericin B supplemented with 15% fetal bovine serum (FBS, Hyclone)) at 37°C with 10% CO_2_. VICs at passage 2–5 were used for all experiments. VICs were seeded onto the polyacrylamide substrates at a density of 2000 cells/cm^2^ and cultured in standard media.

Human mesenchymal stem cells (hMSCs, Lonza) were cultured in mesenchymal stem cell growth medium (Lonza) in a 37°C incubator with 5% CO_2_. hMSCs at passage 11 were used for all experiments and were seeded onto the polyacrylamide substrates at a density of 660 cells/cm^2^.

### Immunofluorescent Staining, Microscopy, and Cell Metrics

After six hours of static or dynamic culture (cyclic strain ∼10% at 1 Hz), cells were fixed and permeabilized on the polyacrylamide substrates with a 5.3% formaldehyde (Ted Pella, prod #18505) and 4 µM Triton X-100 (Calbiochem) solution. The cells were labeled for f-actin (phalloidin, green, Invitrogen) and nuclei were visualized (Hoechst 33342, blue, Invitrogen). Cells were visualized with an epifluorescent microscope (Zeiss) and images acquired with a CCD camera. Images of 20 cells were acquired from each substrate (n = 3 per group, experiment run in duplicate). The resulting images were analyzed using Image J (NIH) for the cell spread area and perimeter, and the shape factor (Eq. 1) was computed.
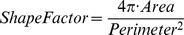
(1)


### Statistics

All values are reported as mean ± standard deviation. Each group consisted of 3 samples. Statistical comparisons were made across all groups (soft, stiff, static, and stretched) using two-way analysis of variance (ANOVA). Differences between groups were determined by post-hoc analysis using the Holm-Sidak method (Sigma Stat). A significance level of 0.05 was used in all the statistical tests performed.

## Results

The protocol for covalently attaching the polyacrylamide to the silicone membranes is relatively straight-forward in theory, but difficult in practice due to multiple critical processing steps. In order to develop a robust protocol to repeatedly produce gels of defined stiffness capable of dynamic stretch, we had to address both the polymerization and covalent attachment of polyacrylamide onto silicone. We found that oxygen plasma is necessary for the covalent attachment, and that both vacuum and nitrogen flow were required to dry the silicone to allow for polymerization. Gels of low (0.3 kPa) and high (50 kPa) shear stiffness (G′) were able to be polymerized in silicone-bottomed culture wells for two different commercially available stretching systems: Flexcell® and STREX. The gels could also be prepared with unmodified fluorescent polystyrene beads; however, we found that modified beads can inhibit polymerization, possibly due to the surface charges (data not shown). We suspect that this process may not work on all silicone as we experienced difficulty polymerizing the gels on the “uniaxial” STREX wells whereas the gels polymerized on the “biaxial” STREX plates; however, the reason is not clear at this point.

Gels polymerized onto silicone membranes had identical appearance as those polymerized on glass. Cells cultured on the polyacrylamide gels had similar responses for both glass and (static) silicone supports. The PA gels attached to silicone membranes can be stretched equibiaxally to 15% at 1 Hz triangle waveform for 12 hours and still remain attached under culture conditions. Longer stretch cycles are currently being investigated. The gels can be fabricated and stored (pre-collagen coating) at 4°C for multiple weeks without any apparent degradation in performance as assessed by visual appearance during manual stretching.

### Strain field transmission

**Figure 2:**
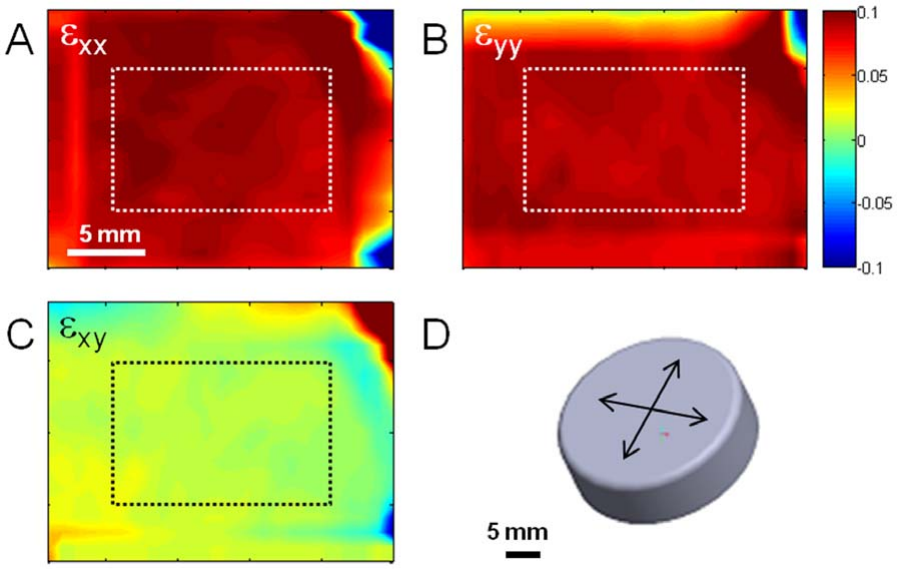
Strain field in region of interest is roughly uniform for equibiaxial stretch. Strain maps for a soft gel (0.3 kPa) undergoing equibiaxial strain in the X (A), Y (B), and XY (shear, C) directions demonstrating relatively homogenous strain and minimal shear within the area of analysis of cell morphology (dashed box). (D) CAD representation of the circular loading platen over which the silicone membrane is stretched by vacuum pressure. Scale bars = 5 mm.

**Figure 3:**
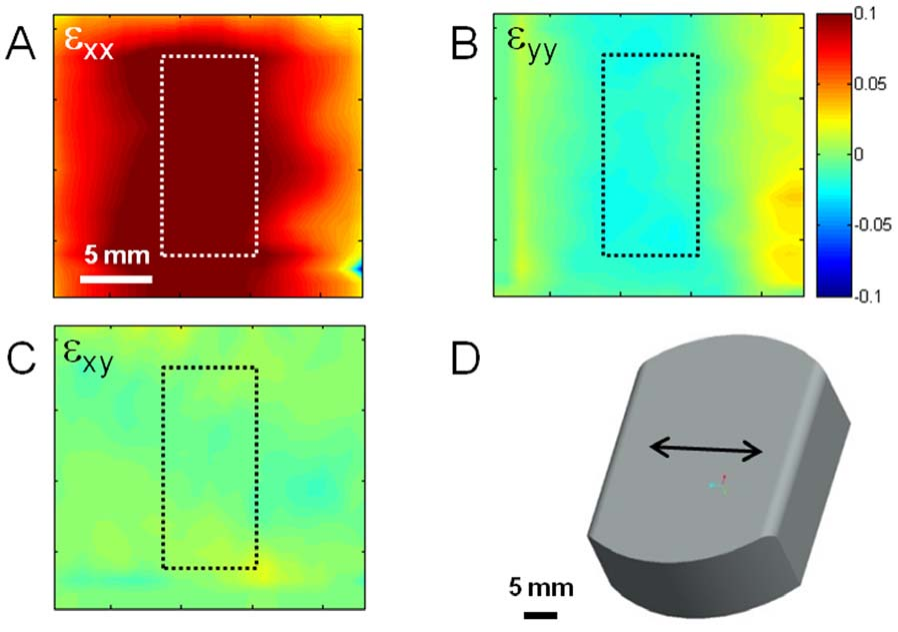
Strain field in region of interest is roughly uniform for pure uniaxial stretch. Strain maps for a soft gel (0.3 kPa) undergoing pure uniaxial strain in the X (A), Y (B), and XY (shear, C) directions demonstrating relatively homogenous strain and minimal shear within the area of analysis of cell morphology (dashed box). (D) CAD representation of the Arctangle™ loading platen over which the silicone membrane is stretched by vacuum pressure. Scale bars = 5 mm.

Strain is transferred through gel and exhibits similar strain patterns compared to unmodified Flexcell® wells although the average value is slightly lower (Figure 2). The lower average strain likely reflects imperfect transfer of strain rather than restriction of membrane deformation due to the presence of the very thin and soft gel (Table 1); however, it is conceivable that the stiff gel may somewhat restrict the motion of the membrane as it is a similar stiffness (50 kPa shear stiffness = 150 kPa Young's modulus if incompressibility is assumed). Alternatively, the treatment with solvents may stiffen the membrane resulting in lower stretch at a given vacuum pressure. The equibiaxal stretch loading posts provide approximately 3.8 cm^2^ homogeneous region in the center (Figure 2). The Arctangle™ loading post produces complex strain field, as expected with roughly pure uniaxial strain in discrete areas along the primary stretch axis (Figure 3).

### Cell culture results

**Figure 4:**
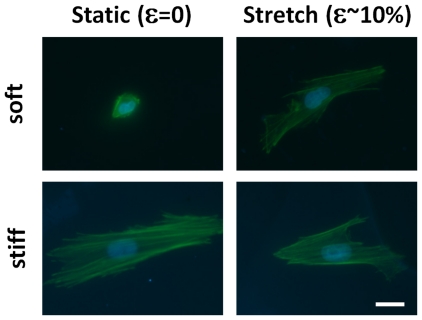
Cells cultured on soft substrate can sense and respond to stretch. Micrographs of valvular interstitial cells cultured statically (left column) and following ∼10% cyclic equibiaxial strain (right column) for 6 hours on soft gels (0.3 kPa, top row) and stiff gels (50 kPa, bottom row). Staining for f-actin (green) and nuclei (blue) shows that stretch increases the spread area of the cells. Scale bar = 20 µm.

**Figure 5:**
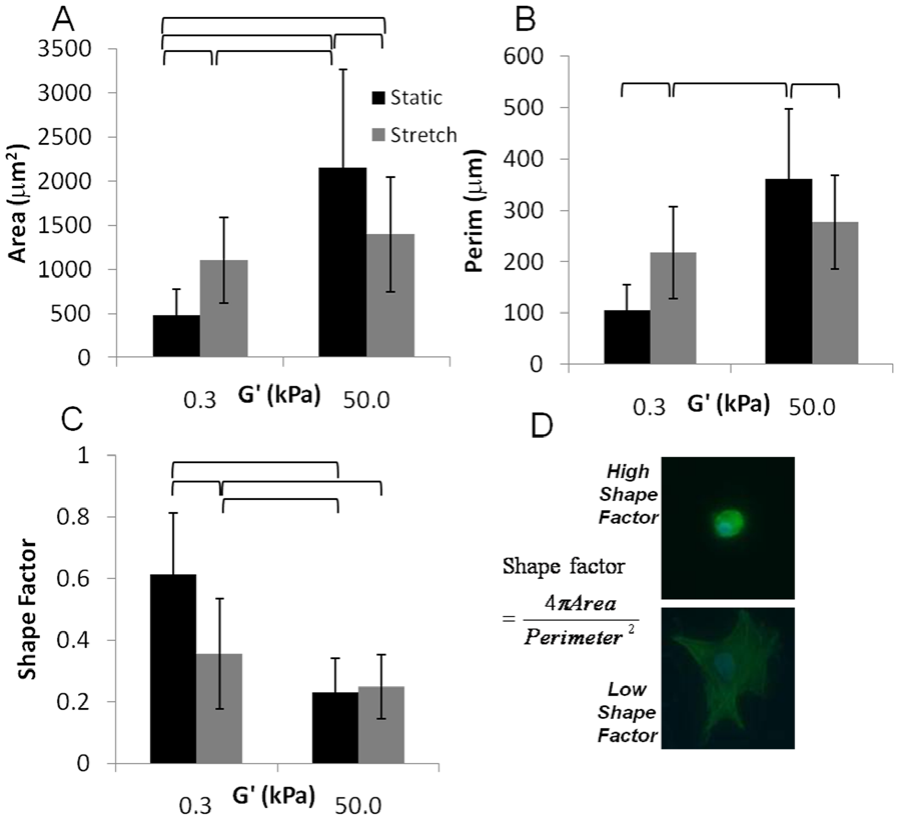
When cyclically stretched, cells on stiff substrates reduce spread area whereas cells on soft substrates increase spread area: Area (A) and perimeter (B) of VICs cultured on low (0.3 kPa) and high (50 kPa) stiffness gels subjected to 10% cyclic stretch at 1 Hz for 6 hours (grey bars) or static (black bars) culture. Shape factor (C) quantifies how rounded a cell is (a shape factor of 1 is perfectly circular, whereas a shape factor of 0 is extremely spread with many extensions). Cells of low and high shape factor are shown in C. Brackets above bars show significance between individual groups (two-way ANOVA, p<0.05).

**Figure 6:**
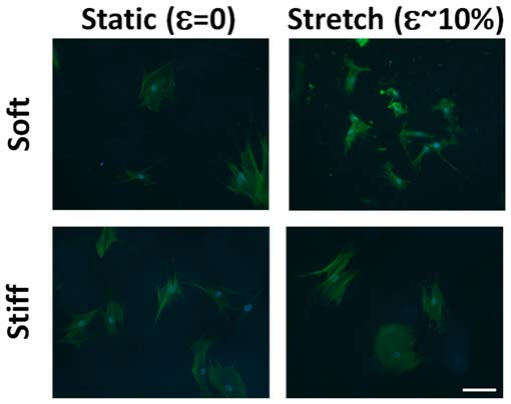
hMSC response to stretch is unclear due to spreading on static soft gels. Micrographs of hMSCs cultured statically (left column) and following ∼10% cyclic equibiaxial strain (right column) for 6 hours on soft gels (0.3 kPa, top row) and stiff gels (50 kPa, bottom row). Staining for f-actin (green) and nuclei (blue) shows that hMSCs on soft gels (static and stretched) have unorganized actin fibers whereas cells on stiff gels have more organized actin fibers. Unlike VICs, hMSCs spread well on soft gels and stretch appears to increase the spread area of the cells slightly on stiff gels. Scale bar = 100 µm.

**Figure 7:**
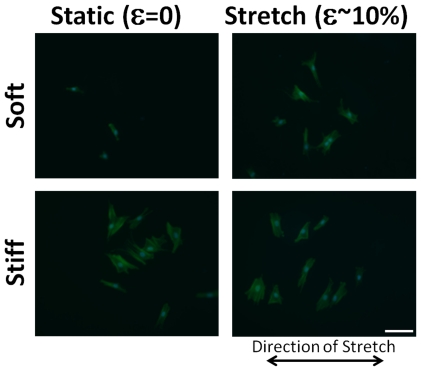
VICs on soft (0.3 kPa) and stiff (50 kPa) gels cultured under static and pure uniaxial stretch conditions (1 Hz, 10% stretch, 6 hrs). Cells cultured on soft substrates appear to have less realignment with stretch compared to the classic realignment perpendicular to the direction of stretch on the stiff substrates. Scale bar = 100 µm.

VICs cultured on static soft gels were small and round and developed pronounced stress fibers with stretch (Figure 4). The spread area of the VICs increased ∼3-fold with stretch of cells on soft gels, but decreased ∼25% for cells on stiff gels with 10% equibiaxial stretch (Figure 5). The spread area of VICs on soft-stretched gels was not significantly different than on stiff-stretched gels (p<0.05), although the perimeter was smaller (p<0.05). The shape factor (function of area and perimeter, indicating relative amount of cellular extension) decreased approximately two-fold with stretch of cells on soft gels and did not change significantly for cells on stiff gels (Figure 5). Stretching hMSCs cultured on soft gels affected cell spread area to a lesser extent (compared to VICs) which was likely due to the ability of hMSCs to spread on lower stiffness substrates (thus little additional spreading occurred with stretch, Figure 6). VICs cultured on soft substrates (0.3 kPa) and subjected to uniaxial stretch showed minimal alignment perpendicular to the direction of stretch, whereas VICs on stiff substrates under the same stretch conditions had pronounced alignment perpendicular to the stretch direction (Figure 7).

## Discussion

In this study, we developed a novel method for combining and independently controlling two important mechanical cues: the stiffness of the culture substrate and dynamic stretch. Our preliminary data confirm our hypothesis that the application of cyclic stretch to cells cultured on soft hydrogels induces responses indicative of culture on stiff substrates; most strikingly, valvular interstitial cells exhibited a rounded morphology on soft static substrates but spread to the same extent as those cultured on stiff substrates upon application of cyclic equibixal stretch. Previous studies have shown that cells remain mechanically sensitive when cultured on soft substrates [22], yet it was unclear *a priori* if rounded cells on very soft substrates would retain mechanosensitivity or even be capable of remaining adhered to the substrates when subjected to large cyclic biaxial strains of extended duration. Our data indicate that cells on soft substrates remain well attached and have functional mechanical sensing mechanisms despite their rounded morphology and low basal tension level and that the application of stretch can override stiffness cues.

### Cell phenotypic modulation and differentiation

Our main purpose for developing the combined stretch and stiffness method was to facilitate the study of mechanical modulation of cell phenotype and differentiation in a more biofidelic mechanical environment. Cells within connective tissues reside in soft environments (relative to tissue culture plastic and silicone membranes) and are dynamically stretched due to external loading of the tissues and traction forces from other cells. We are especially interested in the mechanical regulation of VIC phenotype due to the strong correlation of myofibroblasts and fibrotic pathology in areas of high stiffness and stretch in the valve. The valve leaflet environment is highly heterogeneous with very soft and stiff regions as well as extremely large dynamic strains. Our data indicate that VICs are highly sensitive to combinations of stretch and stiffness. Although determination of phenotypic shifts awaits analysis of functional outcomes such as gene/protein expression and traction force generation, these results may have implications for scaffold design. If a soft substrate is chosen to reduce cell tension and limit fibrotic behavior within a scaffold (to inhibit myofibroblast activation), the magnitude of stretch will be higher than in a stiff scaffold for a given loading, and the stretch stimulus may be sufficient to produce an equivalent (or potentially enhanced) fibrotic behavior as observed with a stiff scaffold.

The method developed herein is potentially applicable to the study of mechanoregulation of any adherent cell. Our experimentation with different cell types indicates that the threshold for responses to stiffness and stretch is likely different for each type of cell (compare Figures 4 and 6). Mechanical regulation of stem cells is currently of great interest, and there is mounting evidence that stem cell lineage is directed, at least in part, by the local stiffness with osteogenic lineage favored on more rigid substrates, adipogenic or neuronal differentiation enhanced on soft substrates, and muscle markers expressed on intermediate stiffness substrates [17], [18]. These findings have practical implications for *in vitro* differentiation of stem cells for cell-based therapies in addition to the fate of the stem cells once implanted. For example, it has been suggested that the heightened stiffness of post-myocardial infarction scar tissue is not conducive to induction of stem cell differentiation to the proper (muscle) lineage [27]. It is conceivable that the cells may even be pushed towards an osteogenic lineage in a stiff scar. Similarly, cyclic stretch has been shown to induce differentiation of bone marrow stem cells into different cell lineages including ligament cells [28], chondrocytes, osteogenic cells [29], myocardial cells, and vascular cells [30], [31] in a stretch anisotropy (uniaxial vs. equibiaxial) [32] and strain magnitude-dependent manner [33], [34]. Although the effect of stretch on spreading of hMSCs (Figure 6) on soft substrates was inconclusive in this study since they did not demonstrate a rounded morphology on the low level stiffness gel, lower stiffness gels could be utilized in the present system (we have attached gels down to 50 Pa). Controlling combined levels of stretch and stiffness simultaneously holds the promise of providing more flexibility in the induction of specific stem cell lineage than stiffness or stretch alone.

### Cell contractility and prestress

The ability of the cell to generate tension through its actin cytoskeleton is integral to the mechanoregulation of cell behavior. For example, stiffness-directed stem cell lineage specification is blocked by inhibiting nonmuscle myosin II [17], and endothelial cell reorientation with cyclic uniaxial stretch is blocked by abolishing stress fibers [35]. Cell traction force is, in turn, strongly modulated by the substrate stiffness [24], [36]. Thus, tunable stiffness substrates offer a powerful alternative to chemical agents for the study of how cell prestress levels alter the transduction of dynamic stretch. More recently, dynamic substrates that utilize UV light to decrease stiffness were developed to evaluate the cellular effects of changes in stiffness in a single substrate during culture [20], [37]. While these dynamic systems allow the study of the transition between multiple levels of stiffness, they do not address the differences in cell signaling between stiffness and stretch. Similar to previous chemical blocking experiments, stress fibers are absent on soft substrates; however, our data clearly demonstrate the ability of the cells to form stress fibers and remodel their cytoskeletons in response to cyclic stretch in the absence of high cell prestress (Figure 4). Not only are the potential side effects of chemical blocking agents removed by using PA gels, the prestress in the cell can be tuned to various levels by selecting the stiffness of the gel, and the traction force before, during, and after stretch can be assessed by utilizing traction force microscopy, a technique widely utilized with standard PA gels [21], [22], [38]. The incorporation of fluorescent microbeads in PA gels cast in between glass plates is relatively straight forward; however, care must be taken when selecting the type of beads as to not affect the polymerization or attachment to the silicone. We have found that beads with surface modifications such as carboxylate groups interfere with the gel polymerization and attachment. Our preferred method for incorporating beads into PA gels cast onto silicone is to first cast a gel (as described above) and once polymerized, apply a thin layer of gel/(unmodified) bead solution on top. Only recently has cell traction forces in response to stretch been evaluated, and it was found that forces initially decreased and then slowly recovered after a single on-off stretch cycle [22].

### Cytoskeletal changes (cell area and stress fibers)

Quantification of cell traction force is also critical for studying the mechanisms by which the cytoskeleton is remodeled in response to both stiffness and stretch. VICs cultured on high stiffness substrates, presumably at a high level of prestress based on their large spread area, actually reduced their spread area when stretched (Figure 4). This behavior has been observed previously with 1 Hz equibiaxial stretch of endothelial cells (but not 0.01 Hz stretch) [6] and is consistent with the stress fiber disassembly and reassembly to remain at a tension set-point. Interestingly, cell retraction was observed without an increase in the rate of disassembly and reassembly of stress fibers in the aforementioned study. Others have observed stress fiber shortening after only one cycle of quasistatic stretch of NIH 3T3 cells [39] and rapid fluidization of the cytoskeleton in human airway smooth muscle cells [22]. Although spreading due to cyclic stretch of cells on a soft substrate has not previously been shown, this behavior (Figure 4) is also consistent with the fibers remodeling and lengthening to reestablish a mean level of fiber tension when extended cyclically. Kaunas and colleagues [6] have developed a model incorporating viscoelastic stress fibers which predicts high tension in the fibers at high strain rates, but a negligible perturbation in fiber tension at the low strain rate consistent with the observed data. Although this and other models have been successful in predicting the dynamics of cell reorientation with uniaxial stretch, cell spreading and retraction are not explicitly predicted by any model to the best of our knowledge.

From a feedback-control system point of view, it is still controversial whether the cell has an extension (strain) setpoint or a tension (stress) setpoint; [40] or possibly it is a hybrid system controlling both the stress and strain state in the cell to control a basal energy level [41]. The feedback loop likely contains chemical diffusion and/or bond formation/dissociation and thus is sensitive not only to differences from the setpoint (proportional control) but also the rate of change of the signal (derivative control) [42] and the summation of signals over time (integral control). Further, the control is most likely nonlinear since the cells can actively adapt to the stimuli. Quantification of cell traction forces, dynamically varying the stiffness of the gel, applying additional non-equibiaxial, non-homogeneous strain patterns, and changing the rate of strain both for loading and unloading will provide new data for validation of computational models and will shed light upon the mechanical control system of the cell.

### Mechanotransduction

The similarity of spread morphology of VICs with application of stretch on soft substrates to those cultured statically on higher stiffness substrates leads us to speculate that the mechanisms of “outside-in” sensing (of stretch) are similar to those for “inside-out” sensing (of stiffness). However, identification of the mechanosensors which transduce substrate stiffness and/or stretch is not trivial since they may be located anywhere along the mechanical pathway from outside the cell, to the interface between the cell and ECM, to deep within the cell. It is likely that there are multiple types of mechanosensors including mechanically actuated protein unfolding [43], stretch-sensitive ion channels [44], and changes in protein kinetics with loading such as actin stabilization [6]. Further, it is difficult to distinguish between inactivation of a mechanical or chemical pathway from inactivation of a mechanosensor itself since the physical linkages necessary for relaying the mechanical signal to the sensor may be disrupted by experimental treatments. For example, blocking integrin expression may disrupt mechanotransduction due to the mechanosensitivity of the integrins themselves, or due to lack of sufficient attachment to the substrate as integrins are the critical for anchorage to the ECM. Independent stretch and stiffness control offers the possibility of separating the effects of outside-in vs. inside-out signaling.

### Other stiffness-stretch methods

Other materials and methods could be used to obtain combined levels of stiffness and stretch. For example, PEG, PDMS, or other soft polymers have been utilized for the study of stiffness-dependent biology and could be integrated into a similar stretch device [45]. Further, beds of microneedles of various dimensions have also been extensively used as tunable stiffness culture substrates [18], [46] and could be stretched, although it is unclear if cells would attach to adjacent posts and spread once adhered to a given set of posts. The thickness of a thin (1–10 µm) collagen gel [47] or PA gel [48] layer attached to a silicone membrane could also be altered to modulate the effective stiffness sensed by the cells if the thickness could be controlled and the gel affixed tightly. For the development of our method, we chose to use a relatively thick layer (70 µm) of the most common tunable-stiffness substrate, polyacrylamide, due to the known conjugation chemistries for various ECM coating proteins and the extensive traction force microscopy methods developed for PA gels. We chose to affix PA onto the most common commercial stretching device (Flexcell®), although we have also affixed PA to other commercial cell stretching devices (e.g., STREX, B-Bridge) and custom devices utilizing silicone sheeting (e.g., Specialty Manufacturing). Alternatively, the previously mentioned elegant indenter-based device for stretching individual cells on PA [22] could be scaled up to stretch larger numbers of cells simultaneously.

### Limitations/Future

As we have shown in this study, the ability to independently control the stiffness and stretch of a 2D culture substrate represents a substantial advance for studies of mechanobiology; however, cells have repeatedly been shown to behave differently in 2D culture than in three-dimensional (3D) systems [49]. The cell shape, motility, proliferation, and protein biosynthesis are often very different in cells cultured on 2D substrates compared to those cultured within 3D synthetic and biopolymer gels. Further, cells cultured within soft biopolymer gels orient towards the direction of stretch [50] whereas the opposite response is found for cells cultured on 2D stiff substrates [51]. This response could be attributed to contact guidance, but could also be a result of the compliance of the gel. Despite these differences, 2D systems remain important for the study of mechanobiology due to the wealth of powerful techniques available to interrogate the cells in 2D and the ability to control other important factors which may affect cell responses including nanotopography and ligand density offered to the cell.

Here we focused on studying relatively large cell populations in parallel for statistical changes and to allow for future gene/protein quantification. Clearly there is a need to integrate the PA layer onto flex units on a microscope stage to track single cell behavior over time (e.g., using the STREX system). Further, dynamic changes in substrate stiffness should be investigated to study their interaction with changes in stretch [20], [37]. Finally, chemical signals are integrated with mechanical signals within the cell, thus combinations of growth factors and mechanical stimuli should be examined in concert in future studies.

In summary, we report on a novel method for the study of mechanobiology which enables independent control of stretch and stiffness of the culture substrate. To facilitate adoption by other research groups, the method combines the most highly utilized tunable-stiffness substrate with the most common stretching apparatus available. Preliminary results demonstrate, for the first time, spreading of rounded cells on soft substrates in response to cyclic equibiaxial stretch. Studies using this method may increase our understanding of mechanical regulation of cell differentiation and phenotype, validate computational models of dynamic cell remodeling in response to stretch, and help elucidate molecular mechanisms involved in mechanotransduction of both outside-in and inside-out signaling.
